# Influence of patients’ living conditions on tuberculosis treatment outcomes in a South African health sub-district

**DOI:** 10.4102/safp.v62i1.5036

**Published:** 2020-08-25

**Authors:** Tombo Bongongo, Hendry van der Heever, Doudou K. Nzaumvila, Christian N.S. Saidiya

**Affiliations:** 1 Department of Family Medicine and Primary Health Care, Sefako Makgatho Health Sciences University, Pretoria, South Africa; 2 Department of Public Health, Health Systems Management and Policy, School of Health Care Science, Sefako Makgatho Health Sciences University, Pretoria, South Africa

**Keywords:** living conditions, tuberculosis treatment outcomes, Tshwane health district, South Africa, public health

## Abstract

**Background:**

Tuberculosis (TB) remains a serious public health concern because it continues devastating communities. This survey was conducted in the sub-district 2 of the Tshwane health district, South Africa. It aimed at determining the influence of patients’ living conditions on TB treatment outcomes. Human immunodeficiency virus (HIV) status, food security and exposure to cigarette smoke were considered as living conditions; and cure, death, default, failure and relapse were considered TB treatment outcomes.

**Methods:**

Record review using the Aitahealth database, clinic registers as well as a piloted, structured and administered questionnaire.

**Results:**

Convenience sampling applied; 180 respondents were obtained. Tuberculosis respondents with negative HIV status had a cure rate of 67.3% whilst those with positive HIV status had 37%. Tuberculosis respondents with good food security had 45.9% of cure rate. Tuberculosis respondents exposed to cigarette smoke had a death rate of 65.2%, while respondents not exposed to cigarette smoke showed 75% of cure rate.

**Conclusion:**

HIV status, food security and exposure to cigarette smoke, as components of living conditions, showed an association with TB treatment outcomes in the selected sample; in the sense HIV infection reduced the cure rate, increased the death and default rates of TB patients in the same sample. Good food security increased the cure rate of TB patients, but exposure to cigarette smoke decreased the cure rate and increased the death rate amongst respondents having TB treatment in the current survey.

## Overview

Despite all efforts made by the World Health Organization (WHO), such as the six-point Stop Tuberculosis (TB) Strategy to reach the global targets, and the joint efforts by WHO and United Nations programme on HIV/AIDS (UNAIDS), as stipulated in one of the 13 targets of the sustainable development goals that explicitly talks about the end of TB by 2030,^1^ this disease still remains a serious public health issue nationally as well as globally.^2,3^

The WHO report of 2018 refers to about 10 million people having TB and 1.3 million deaths because of TB worldwide in 2017. Considering deaths because of TB with human immunodeficiency virus (HIV) as co-infection during the same year, 300 000 more deaths have to be added to the WHO count.^1^

An estimated 124 000 people died of TB in South Africa in 2016, with an average of 330 deaths per day. Tuberculosis remained the leading cause of death. Its association with HIV co-infection has aggravated the situation. Over 80% of those who died as a result of TB were HIV-positive.^4^

The South African National TB Guidelines 2014 define ‘cure, death, default and relapse’. Cure applies to the individual who had a positive TB smear or culture at diagnosis, which becomes negative at least on one instance 4 weeks preceding the end of the treatment as well as at the end of the treatment. ‘Death’ refers to a patient who dies whilst taking anti-TB treatment, regardless of the cause of death. ‘Default’ is used for a patient who has taken at least 1 month of anti-TB treatment and interrupts the same treatment for 2 months or more. ‘Treatment failure’ is used for the individual on TB treatment, who remained on or converted to positive smear or culture before the end of therapy. ‘Relapse’ is used when a TB patient who has been cured develops the disease at the end of the treatment period.^5^

Outcomes of TB treatment could be influenced by factors such as living conditions, referred to as circumstances or factors affecting the way in which people live, especially regarding their well-being.^6^ For the purpose of this study, HIV status, nutrition and exposure to cigarette smoke are considered. Human immunodeficiency virus infection as a health condition with strong association and influencing the well-being of TB patients cannot be ignored. The living conditions of TB patients, as described by various authors, seem to be depressing.^7^ This observation has been verbalised, on numerous occasions, by the sub-district 2 community health workers (CHWs) and served as a strong motivation regarding the initiation of the current survey.

### Considering human immunodeficiency virus status

Human immunodeficiency virus and TB infection have increased in prevalence worldwide, as confirmed by some of the researchers in the United Kingdom (UK). Being HIV-positive appears to be a predisposing factor for developing TB. Statistics have indicated that more TB patients are expected based on the fact that HIV prevalence has increased. Tuberculosis could be the leading cause of death in many HIV-related deaths. Owing to the opportunistic infections that occur in HIV-positive people, mortality would remain high even if patients are put on treatment for TB as well as HIV. Tuberculosis and HIV infection have to be considered seriously by all stakeholders in order to prevent development of an irreparable situation.^8^

In rural Nigeria, HIV co-infection and others factors such as multiple drug-resistant TB were considered as predictors of unsuccessful TB treatment. Unsuccessful TB treatment outcomes could be avoided by emphasising a close monitoring of TB patients and also by offering socio-economic assistance to patients as well as promoting activities against the spreading of HIV.^9^

Although a cure rate of 70% was reached in other parts of Africa whilst treating TB patients with HIV co-infection, this treatment success rate is below the WHO-laid target of 90% as recommended in the Global Plan to Stop TB by 2015.^10^

A study which looked at the factors associated with unsuccessful TB treatment outcomes amongst TB patients also being HIV-postive was conducted in Ethiopia. Factors identified included living outside the Gondar town in Ethiopia, weighing less than 43.7 kg at the beginning of TB treatment, being bed-ridden and complaining about the side effects of TB treatment. The study assisted in tracing the factors associated with unsuccessful treatment in order to strengthen the management, rather than to improve the treatment of TB patients co-infected with HIV.^11^

Amongst the TB population in Cape Town, half of the respondents were also HIV-positive. One of the objectives of this study was to identify risk factors associated with TB treatment outcomes. Two groups of TB respondents were compared and the results showed a high rate of death and a low rate of treatment completion amongst the participants co-infected with HIV. It was acknowledged in the study that the burden of TB and HIV infection has posed a worry for the community. Tuberculosis participants who were also HIV-positive had a higher mortality and a lower treatment completion rate compared to TB respondents who were not HIV-positive.^12^

### Considering food security (had sufficient food on a daily basis for the past 12 months)

A study from Afghanistan has found that poverty, food insecurity and poor nutrition could contribute to the occurrence of TB. To overcome situations that negatively impact the TB treatment outcome, a plan was initiated to assist TB patients to gain better adherence to the treatment. The World Food Programme provides an incentive to TB patients who are enrolled in the national TB programme. This initiative assists with good adherence to the treatment and also boosts the challenging nutritional status of patients as well as that of their households. This initiative has led to the WHO’s successful Directly Observed Treatment, short-course (DOTS) in the country.^13^

A randomised controlled trial was conducted in South Africa with TB respondents divided into two groups: the first group with financial support, and the second one without financial support. Knowing that poverty affects adherence to treatment and negatively impacts the TB treatment outcome, economic support in the form of a voucher was offered to participants in the intervention group. Both groups received TB treatment. The target was to assess whether economic support would assist community in terms of reducing TB burden. The economic intervention, comprising R120.00 (South African currency) per month, was provided to each of the 110 patients to enable them to buy food. As a result, although there is a strong significance between the availability of food and the success of TB treatment, the study did not establish a relationship between economic support to TB patients and a better TB treatment outcome.^14^

### Concerning exposure to cigarette smoke (active or passive)

The effect of smoking on the treatment outcomes amongst TB patients was studied in the Philippines. The study was motivated by the fact that not much has been written on TB treatment outcomes versus smoking. It is known that smoking increases the prevalence of TB. This study aimed to establish the association between smoking and TB treatment outcomes. The study was conducted in a hospital where DOTS was applied. In conclusion, the study showed an association between smoking and TB treatment failure.^15^

India is known to have the highest TB prevalence rate and cigarette smoking habits. Based on two parameters, a study was conducted amongst new TB patients, regarding the effects of smoking on the treatment outcomes. Respondents were grouped based on whether they never smoked, were currently smoking or were ex-smokers. Amongst respondents who were smoking and those who were ex-smokers, the bacteriological control remained positive after 8 weeks of treatment initiation. These two groups of new TB patients presented a substantially high rate of defaults, treatment failures and relapses. Smoking was also associated with the risk of contracting a severe form of disease and a slow bacteriological conversion.^16^

In South Africa, a study was conducted that confirmed that tobacco smoking could be associated with the occurrence of cancer, heart diseases as well as TB. It was a retrospective cohort that assessed outcomes of TB treatment amongst tobacco users. It established the baseline characteristics that could be associated with the use of tobacco as well as the population of tobacco users having TB. Data collected and analysed showed that smoking is one of the societal behaviours associated with the emergence of TB that also adversely affects TB treatment outcomes.^17^

In a study conducted in Cape Town, South Africa, on how smoking slows down TB healing^18^, highlighted the fact that TB treatment outcomes and tobacco smoking seem to be a major public health problem. Many TB patients, who smoked, had their sputum culture conversion delayed. By the end of the first 2 months of TB treatment, also called the intensive phase, all patients should have a negative sputum culture. It appeared that actively smoking participants did not have converted sputum culture at this stage, compared with non-smokers. This would often result in the extension of duration of TB treatment and unsuccessful treatment outcomes. The study showed that quitting cigarette smoking when commencing TB treatment was strongly recommended. This recommendation, once applied, could assist to improve the TB treatment outcomes.^18^

Looking at the fact that not much has been described on the influence of patients’ living conditions on TB treatment outcomes, specifically in the Tshwane health district, this current survey aimed at determining the influence of patients’ living conditions (i.e. HIV status, food security and exposure to cigarette smoke) on the TB treatment outcomes (cure, death, default, failure and relapse) in a South African health sub-district (Tshwane).

## Methods

### Study design

The study design comprised record review using the Aitahealth database, clinic registers as well as a piloted, structured and administered questionnaire. A pilot study was carried out on 20 patients at the Phedisong 4 community health centre (CHC), located in sub-district 1 of the Tshwane health district. The ward-based outreach team questionnaire was adapted in the revised questionnaire compiled by the researcher.

### Setting

The survey was conducted in sub-district 2 of the Tshwane health district. It is located next to sub-district 1 in the northern part of the Gauteng Province, South Africa. It comprises one CHC and 11 clinics; of these, only the CHC and eight clinics were considered in this survey. Clinics were selected based on their geographic accessibility and the availability of a fully trained and functional ward-based outreach teams.

### Questionnaire

The questionnaire had six groups of questions related to the diagnosis of TB, demographics, HIV status, nutrition, exposure to cigarette smoke and the TB treatment outcomes. The questionnaires were written in English and Se-Tshwana since these are the two spoken languages in sub-distruct 2 of Tshwane. The questionnaire (English version) was compiled by the researcher. The Se-Tshwana translation was done by a medical doctor conversant in both English and Se-Tshwana. Adjustments were firstly made by the researcher, then by two family physicians (co-authors) and finally by a senior expert in research.

### Data collection

The researcher trained a CHW as research assistant. He was able to express himself in the two spoken languages of the area (English and Se-Tshwana). He was tasked to collect data. With the information obtained from the TB registers of the nine clinics of sub-district 2 and from the Aitahealth database (a database having all ward-based outreach team (WBOT) information of patients in Tshwane, such as diagnosis and physical address). The research assistant went from house to house to collect raw data either from TB patients or from the relatives of deceased patients. Looking at the TB treatment outcomes and the HIV status, although this information was recorded in clinics’ TB registers, respondents were asked to confirm their status. Their responses were cross-checked with the information obtained from clinic registers. In case of discrepancy, respondents were encouraged to go back to the clinic for clarity and assistance. For the purpose of this study, the clinic results were used rather than patients’ responses. However, most of the patients knew the outcome of the treatment and their status. Regarding exposure to cigarette smoke, the study included both active and passive smokers. Respondents who were not smoking or did not have a past history of smoking and were not living with a smoker were classified as not-exposed.

The respondents were thoroughly informed regarding the survey. It was only after full understanding that these respondents or relatives of the deceased were advised to consent for the study and the questionnaires were administered.

### Inclusion and exclusion criteria

All TB patients registered with the nine clinics of sub-district 2 of the Tshwane health district, as well as with the WBOT, were included. All patients younger than 18 years of age were excluded.

### Study population and sampling

From the Aitahealth database and the cross-checking performed with the eight clinic registers, plus 1 CHC register, a total of 193 TB patients were considered. However, some of the patients had changed their addresses and could not be located and a few relatives of the deceased refused to provide information on deceased because of bad memories it evoked. Therefore, it was reasonable to opt for convenience sampling whilst recruiting respondents. A total of 180 questionnaires were completed.

### Data analysis

The raw data were transferred unto a Microsoft Excel spreadsheet and statistical analyses was performed using SAS (SAS Institute Inc, Carey, NC, United States), version 9.4. Associations were tested for significance using Fisher’s exact test. The Fisher’s exact test calculates exact *p*-values and is suitable for small samples, whereas the chi-square test calculates approximate *p*-values and is suitable for large samples. In this study, *p* ≤ 0.05 was considered significant.

### Ethical consideration

A clearance certificate 179 was granted by the Sefako Makgatho Health Sciences University Research Committee (SMUREC/H/150/2016: PG), and permission to collect data from sub-district 2 of Tshwane health district was granted by the Tshwane health district authority. Written consent for taking part in this survey was obtained from all the respondents; they were informed about their rights to withdraw at any time from the survey if they experienced any discomfort with discussions.

### Results

In terms of respondents, there were more male respondents (*n* = 107; 59.4%) than female respondents. More respondents (*n* = 107; 59.4%) had a secondary level of education, and more were employed (*n* = 93; 51.7%; Table 1).

**TABLE 1:** Baseline characteristics (*n* = 180).

Variables	Frequency (*n*)	%
**Gender**		
Male	107	59.4
Female	73	40.6
**Education**		
None	16	9.0
Primary	49	27.2
Secondary	107	59.4
Tertiary	8	4.4
**Employment**		
Unemployed	87	48.3
Employed	93	51.7

Of 180 respondents, 46.1% had a positive HIV status. The majority of them (*n* = 124; 68.8%) had sufficient food on daily basis for the past 12 months. The majority of respondents (*n* = 111; 61.7%) were not exposed to cigarette smoke (Table 2).

**TABLE 2:** Living conditions (*n* = 180).

Variables	Frequency (*n*)	%
**HIV**		
Positive	83	46.1
Negative	49	27.2
Unknown	48	26.7
**Food security**		
Good	124	68.8
Bad	56	31.11
**Cigarette smoke exposure**		
Exposed	69	38.3
Not exposed	111	61.7

HIV, human immunodeficiency virus.

### Human immunodeficiency virus status and tuberculosis treatment outcome

More HIV negative participants (*n* = 33; 67.4%) were cured; more deaths (*n* = 22; 26.5%) and defaults (*n* = 26; 31.3%) were noted amongst HIV-positive patients. Association between TB patients on treatment who were cured, died or defaulted, when compared with their HIV status, was indicated by *p* = 0.001, 0.02 and 0.03 respectively.

### Food security and tuberculosis treatment outcome

More participants (*n* = 124; 68.8%) were able to have at least one meal per day for the past 12 months. In all, 57 participants (45.9%) amongst those who had food were cured. Whilst comparing the TB treatment outcomes and food security, an association of statistical significance in the group of relapses was noted, with *p* = 0.029.

### Exposure to cigarette smoke

The effect of exposure to cigarette smoke in respondents’ families (passive or active) was evident. Of the 180 respondents with TB, 75 (67.6%) of the respondents who were not exposed to cigarette smoke were cured (*p* = 0.0001), whilst 45 (65.2%) of the respondents who were exposed to cigarette smoke died.

## Discussion

The current survey demonstrates a high rate of HIV-positive status amongst TB patients, which is in keeping with what has been described in numerous countries, including the UK. Owing to the intriguing association between TB and HIV, the expectation, in the UK, was to see more people infected with TB as a result of an increased HIV prevalence in the world.^8^ Such an expectation can also be applied in South Africa, where the number of people living with HIV has increased from 4.25 million in 2002 to 7.52 million in 2018.^19^ The UK study also showed a higher mortality rate amongst TB patients who were also HIV-positive, supported by the current survey (26.5%), as shown in Table 3.

**TABLE 3:** Comparison of tuberculosis treatment outcomes with human immunodeficiency virus status (*n*= 180).

HIV status	Tuberculosis treatment outcomes					
	Cured	Death	Default	Failure	Relapse	Total
**Positive**						
*n*	31	22	26	3	1	83
%	37.35	26.51	31.33	3.61	1.20	-
**Negative**						
***n***	33	5	7	2	2	49
%	67.35	10.20	14.29	4.08	4.08	-
**Unknown**						
*n*	22	19	4	3	0	48
%	45.83	29.58	8.33	6.25	00.00	-
*p*-value, positive vs. negative	0.0011	0.0269	0.0372	1.0000	0.5549	-

HIV, human immunodeficiency virus.

Although poor nutrition and food insecurity contributed to the prevalence of TB in Afghanistan,^13^ the Tshwane survey has demonstrated an association between food security (good food security) and relapse as a TB treatment outcome. Tuberculosis treatment outcome showed very little difference between where food security was and where there was little or no food security. For instance, with food security the death rate was 27.45% and the default rate was 20.1%, but without food security the death rate and the default rate were the same, that is, 21.4%.

By reducing the cure rate and increasing the death and default rate, HIV infection seems to appear, in this current survey, as a predictor of an unsuccessful TB treatment outcome. This finding is in keeping with the outcome of a Nigerian study.^9^ As a rescuing strategy, an intervention programme, such as monitoring helpless groups and promoting socio-economic support as well as action against TB and HIV infection, was planned.^9^

An Ethiopian study showed a 70% TB treatment success rate amongst TB patients with HIV co-infection,^11^ whilst the current Tshwane survey showed that 37.3% TB patients with HIV were cured, as presented in Table 3. Although the goal of 90% set by the WHO for a successful TB treatment^10^ was not reached in both cases, the Ethiopian effort needs to be highlighted and applauded.

A randomised controlled trial on financial support to a group of 256 TB patients, opposed to another group without support, was conducted in South Africa.^14^ The financial support given to the participants of one group was not associated with a better TB treatment outcome. Table 4 shows that there was no significant difference in the cure rate for participants with good food security and those without food security.

**TABLE 4:** Comparison of tuberculosis treatment outcomes by food security (*n*= 180).

Food security	Tuberculosis treatment outcomes					
	Cured	Death	Default	Failure	Relapse	Total
**Yes**						
*n*	57	34	25	8	0	124
%	45.97	27.42	20.16	6.45	00.00	68.8
**No**						
*n*	29	12	12	0	3	56
%	51.79	21.43	21.43	00.00	5.36	31.1
*p*	0.5207	0.4626	0.8442	0.0591	0.0290	-

**Total**	**86**	**46**	**37**	**8**	**3**	**180**

An association between smoking and TB treatment outcomes was studied in the Philippines.^15^ Smoking was shown to be associated with TB treatment failure; but in this current survey, the association was aimed at explaining high death and low cure rates.

The current South African survey did not look at the bacteriological aspect of TB as was the case in India; however, smoking was compared with TB treatment outcomes in both countries. Whilst the Indian study showed an association with default, treatment failure and relapse (high rate of default, failure and relapse), the current South African survey presented an association with cure and death. In essence, smoking increased the death rate and reduced the cure rate in South Africa (Table 5).

**TABLE 5:** Comparison of tuberculosis treatment outcomes by exposure to cigarette smoke (*n*= 180).

Cigarette smoke exposure	Tuberculosis treatment outcomes					
	Cured	Death	Default	Failure	Relapse	Total
**Exposed**						
*n*	11	45	12	1	0	69
%	15.9	65.2	17.4	1.5	0.0	-
**Not exposed**						
*n*	75	1	25	7	3	111
%	67.6	0.9	22.5	6.3	2.7	-
*p*	0.0001	0.0001	0.4527	0.1562	0.2869	-

**Total**	**86**	**46**	**37**	**8**	**3**	**180**

High death rate and low treatment completion rate were the conclusions of a study conducted in Cape Town amongst TB patients who were also HIV-positive.^12^ This Tshwane survey shows a high death rate (26.5%) and also a high default rate (31.3%) amongst TB respondents that are HIV-positive. Whereas amongst HIV-negative TB respondents, the death rate was 10.2% and the default rate was 14.2%, the two studies had reached the same outcomes, that is, high death rate and high default rate for people infected with both TB and HIV.

In Cape Town, as well as in India, it was demonstrated that smokers can complete the intensive phase of TB treatment without having sputum conversion. This may lead to an extension in the duration of treatment.^16,18^

## Conclusion

Human immunodeficiency virus status, food security and exposure to cigarette smoke, as components of living conditions, showed an association with TB treatment outcomes in the selected sample. Human immunodeficiency virus-positive participants showed a reduced cure rate and increased death and default rates in the same sample. Although good food security increased the cure rate of TB patients, exposure to cigarette smoke decreased the cure rate and increased the death rate amongst respondents on TB treatment, according to the present survey.

### Recommendations

At the time of starting the treatment, patients should be educated regarding the influence of HIV, smoking and healthy diet on the outcomes of TB treatment. Social support, such as grant, should also be considered whenever it is possible and appropriate.

### Strengths

Except for the deceased, where the data were provided by third parties, almost all the data were primary data. The questionnaire was piloted and revised by the researcher, peers and the supervisor before it was applied in sub-district 2.

### Limitations

Because verbal information relies on the honesty of respondents and relatives of deceased during the interview, the current survey cannot pretend to present accurate information.
